# Submicron-scale broadband polarization beam splitter using CMOS-compatible materials

**DOI:** 10.1038/s41598-017-05019-3

**Published:** 2017-07-03

**Authors:** Ming-Sheng Lai, Chia-Chien Huang

**Affiliations:** 1 Department of Physics, National Chung Hsing University, 145, Xingda Rd., Taichung, 402 Taiwan, R.O.C.; 2 Institute of Nanoscience, National Chung Hsing University, 145, Xingda Rd., Taichung, 402 Taiwan, R.O.C.

## Abstract

We propose a polarization beam splitter (PBS) with a footprint of only 600 × 790 nm^2^ operating at a wavelength of λ = 1550 nm, which is the smallest PBS ever demonstrated. This device uses CMOS-compatible materials, namely, silicon and silica. The present PBS comprises two Si waveguides with different geometrical aspect ratios adjoined side-by-side, which separates the transverse-electric (TE) and transverse-magnetic (TM) modes without relying on an additional coupling region. The designed PBS achieves a polarization extinction ratio of approximately 25 dB for both modes and insertion losses of approximately 0.87 and 1.09 dB for the TE and TM polarizations, respectively. Over a wide bandwidth of 150 nm (from λ = 1475–1625 nm), a high polarization extinction ratio (greater than 20 dB) and a low inversion loss (lower than 1.3 dB) can be obtained. The proposed PBS allows for geometrical errors of ±15 nm while maintaining a polarization extinction ratio of >20 dB and inversion losses of >1.1 and 1.3 dB for the TE and TM modes, respectively. With the submicron footprint, the reported PBS may be able to be used in high-density photonic integrated circuits and nanophotonic devices.

## Introduction

For improving the operating speed of electronics in integrated circuits due to the RC delay, photonics offers an effective solution to build photonic integrated circuits (PICs) in optical communications. Among photonic components, polarization division multiplexing (PDM) is an efficient solution for manipulating optical signals in PICs, and this allows it to satisfy the ever-increasing demands of signal transmission bandwidths in optical communication systems^1–4^. Among the PDM devices, polarization beam splitters (PBSs), which separate transverse-electric (TE) and transverse-magnetic (TM) polarizations, are the most important devices. They allow the two polarizations to be processed independently, thereby enabling the traffic bandwidth to be doubled. When assessing PBSs, one needs to look at criteria such as device compactness, polarization extinction ratio (PER), insertion loss (IL), operating bandwidth, structure complexity, and fabrication tolerance. The most crucial factor in developing next-generation ultradense PICs in optical communication systems and nanophotonic devices may be the minimization of the PBS areas while retaining satisfactory device performance. Numerous types of PBS devices have been reported^5–30^, including mode evolution (ME)^5, 6^, multimode interference (MMI)^7–10^, Mach–Zehnder interferometer (MZI)^11–13^, photonic crystal (PhC)^14–16^, grating^17–19^, and directional coupler (DC)-based devices^2, 20–30^. In order to reduce the size of the device sizes, silicon-on-insulator (SOI) platforms that have a high-index-contrast property have been adopted in many studies^6, 9, 10, 13, 17–30^. In terms of fabrication, forming photonic devices on SOI platforms is compatible with mature complementary metal-oxide semiconductor (CMOS) technology, which can help to accelerate the development of chip-scale PICs.

With a satisfactory PER of 22 dB, ME-based PBSs are fairly long (>200 μm) and so can adiabatically evolve the field profiles along waveguides, which gives them a better fabrication tolerance and a broad bandwidth of roughly 300 nm^5, 6^. MMI-based PBSs^7–10^, have advantages in terms of fabrication processes and tolerances because of the large areas, making them attractive. However, the dimensions of MMI devices are determined by the common multiples of the self-imaging lengths of the TE and TM modes^31^, which can result in extremely long devices (>1000 μm)^7^. Some innovative MMI-related designs have been reported that can improve the compactness of these devices, including cascaded-types devices (<950 μm)^8^, metal–insulator–metal (MIM)-embedded devices (~44 μm)^9^, and two-mode interference (~8.8 μm) devices^10^. MZI-based PBSs are very long (300–3000 μm)^11–13^,as they modulate the refractive indices of two arms in order to form two asymmetric optical paths. However, their design is very flexible, because it allows for various modulated mechanisms. PhC-based PBSs are formed by the periodic arrangement of dielectric materials^14–16^, and they possess band structures for optical waves, like atomic lattices, for at least one polarization. Therefore, PhC-based PBSs are a few tens of micrometers wide. However, it would be difficult to reduce them further, as they would require enough large extent in order to mimic the periodic environments, and the fabrication processes would be particularly complex. The advantages of grating-based PBSs are that they can simultaneously couple the TE and TM modes by using a single grating waveguide and that they have a fairly high PER of approximately 20 dB^17–19^. Grating-based PBSs are in the order of tens of micrometres; thus, they are difficult to be integrated into planar PICs. The most compact PBSs are the DC-based ones^2, 20–30^, which have device lengths of several micrometers and have satisfactory PERs (10–20 dB). In addition, the simple structure and variety of designs of DC-based PBSs make them the most popular type of PBS. However, their operating bandwidths are relatively narrow, because they utilize precise tuning of phase-matched conditions in order to match specific wavelengths. In principle, a DC-based PBS separates one desired mode by coupling its evanescent field to the cross channel, while maintaining the residual mode propagating along the through channel. Choosing significantly polarization-dependent materials would therefore be helpful in improving the PER and shortening the coupling length of these devices.

Another approach for obtaining high PERs is to excite the surface plasmon polariton (SPP) modes^32^. Furthermore, the larger difference between the effective refractive indices of the TE and TM modes can effectively shorten length of a device. Chee *et al*. adopted a hybrid plasmonic waveguide (HPW)^33^ as the middle waveguide in a three-core arrangement^21^. The length of this design was 6.5 μm, and its PER and IL were greater than 15 dB and less than 0.5 dB, respectively^21, 33^. Guan *et al*. proposed an asymmetrical DC with a device length of 3.7 μm consisting of a Si HPW and Si nanowire^23^; they found its PER to be >12 dB and its operating bandwidth to be about 120 nm. They subsequently reported on a PBS device with a length of 2.5 μm^24^, which was based on a MMI coupler with a Si HPW; however, its PER was limited to being about 10 dB over a bandwidth of 80 nm. Tan *et al*. used nanoscale silver cylinders as the polarization selector between two the Si waveguides of a DC-based PBS device^25^; it was reported to have a length of 1.1 μm, a PER >22 dB, and IL of <0.4 dB. However, the numerical calculations in their study were limited to a two-dimensional structure. Kim and Qi placed a copper nanorod array between two Si waveguides^29^; using a localized SPP, the TE mode of this device was effectively coupled to the cross channel, which reduced the device length to around 2.1 μm and yielded a PER of roughly 15 dB over a broad band of 280 nm. In our previous design^34^, we proposed a novel PBS based on a combined HPW structure. By not having an additional coupling region (in a similar fashion to that of conventional DC-based PBSs), our designed device was able to shortened to 920 nm while exhibiting a PER of around 18 dB and an IL of about 0.6 dB over a broad band of 400 nm. However, connecting the output ports to other Si waveguides moderately degrades the performance of this device. Recently, Zhang *et al*. proposed a graphene-based asymmetrical DC structure composed of a Si waveguide and a graphene multilayer-embedded Si waveguide^35^. The graphene was used so as to replace the metal-forming SPP modes, but it proved to be far more flexible as it could modulate the chemical potential through electrical or chemical approaches. This PBS was 8.3 μm long and had a 200 nm bandwidth and a PER and IL of 18.2 and 0.16 dB, and 21.2 and 0.36 dB, for the through and cross ports, respectively.

In this paper, we report on an ultrashort PBS based on a SOI platform that is compatible with CMOS fabrications. In addition, the high power dissipation of Ohmic losses and the requirement to connect to other Si waveguides from plasmonic waveguide structures were avoided through the use of the all-dielectric materials of Si and SiO_2_ as a way to significantly reduce the ILs. Using two slanted Si waveguides with different geometrical aspect ratios adjoined side-by-side, the TE and TM polarizations were able to be effectively separated without relying on an extra coupling region. Therefore, this design is able to create a device length that is of the submicron scale while still maintaining a satisfactory performance level and broad operating bandwidths. The fabrication tolerances are also discussed in detail in order to assess the feasibility of our designed PBS.

## Results

### Mode properties of the designed PBS

**Figure 1:**
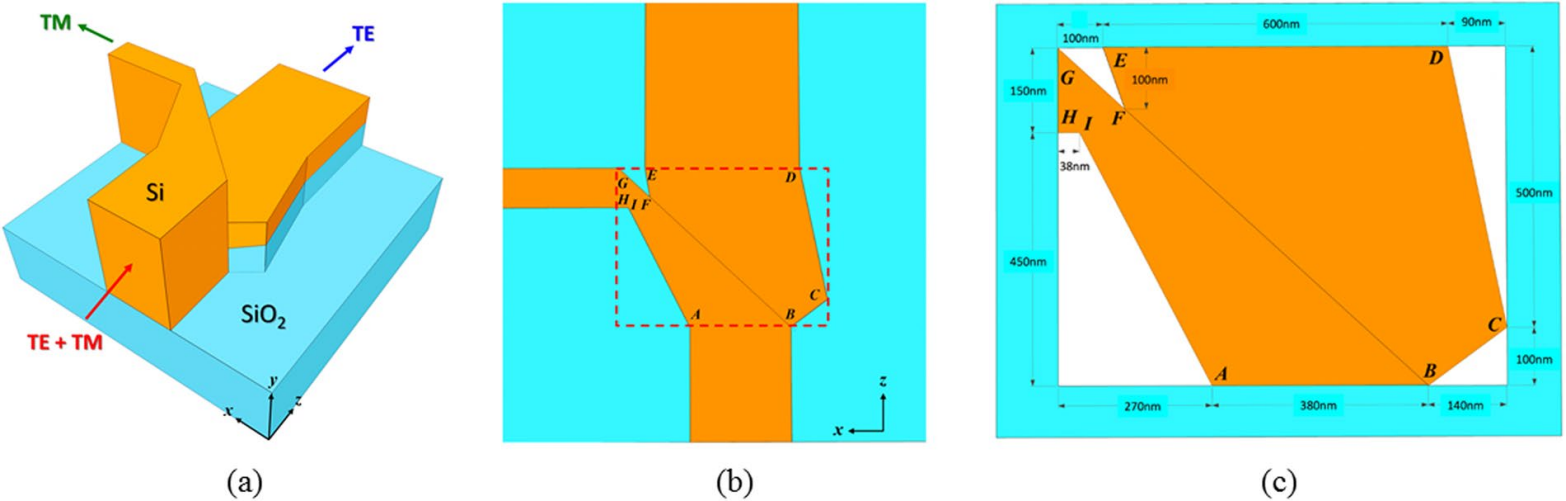
(**a**) A 3D schematic diagram of the proposed PBS, which consists of two slanted Si waveguides with different aspect ratios adjoined side-by-side and deposited on a SiO_2_ substrate; the input (launching the TE and TM modes simultaneously) and the two output (separating the TE and TM modes) channels connected to the PBS are also shown. (**b**) A view from above the proposed PBS, where the closed dashed line denotes the device extent. (**c**) The zoomed-in view of the device extent shown in (**b**).

A 3D schematic diagram of the proposed PBS with connecting input and two output ports is shown in Fig. 1(a). The view from above the PBS and a zoomed-in version that clearly illustrates the dimensions of the device in detail are shown in Fig. 1(b,c), respectively; the device area (790 nm × 600 nm) in the *x–z* plane is indicated by the closed dashed line. The proposed PBS is deposited on a SiO_2_ substrate (blue), and it consists of two slanted Si waveguides (orange) joined ($\overline{BF}$ in Fig. 1(c)) side-by-side; these waveguides have different aspect ratios. In the input port, the height (in the *y*-direction) and width (in the *x*-direction) are 625 and 380 nm ($\overline{AB}$), respectively; this is they can support the fundamental TE (*i.e*., the majority of the electric field is in the *x*-direction) and TM (*i.e*., the majority of the electric field is in the *y*-direction) polarizations, where the definition of the field orientation is relative to the plane of incidence (*y–z* plane).

The front Si waveguide (enclosed in $\overline{ABGHI}$) guides the input TM mode along the gradually tapered bar, and the width ($\overline{GH}$) and height of the output TM port are shrunk to 150 nm (so as to cut off the TE power) and 625 nm (so as to maintain TM power), respectively. However, the input TE power is directly coupled to the Si waveguide (enclosed in $\overline{BCDEF}$) adjoined to the front Si waveguide without an additional coupling length that would otherwise be used in conventional DC-based PBSs^2, 20–30^, which makes the proposed PBS device extremely short. The output TE port is designed to 175 nm high so as to cut off the TM power and 600 nm wide ($\overline{DE}$) so that the TE power can be as high as possible.

**Figure 2:**
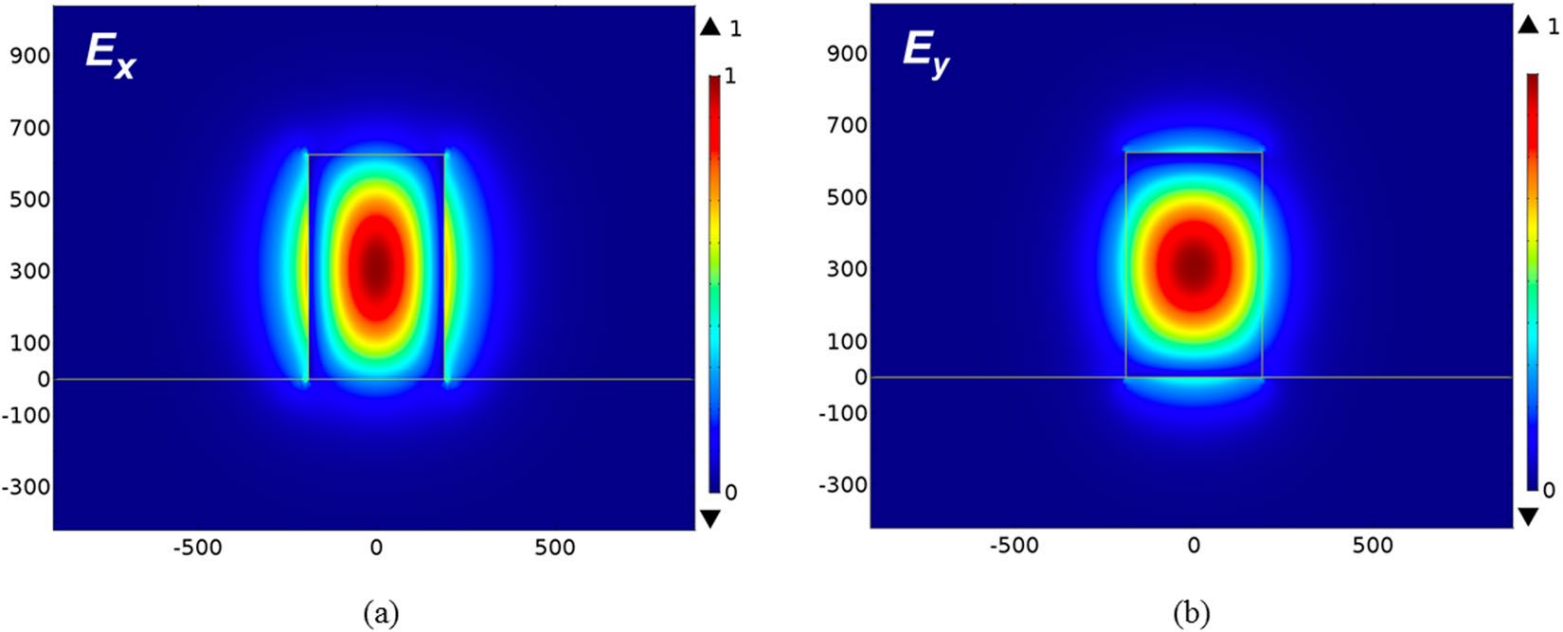
The guided mode profiles of the (**a**) TE (the majority component of electric field, *E*_*x*_) and (**b**) TM (the majority component of electric field, *E*_*y*_) modes at the input port.

The fabrication processes of the proposed device are described as follows. (1) A Si substrate is prepared for depositing SiO_2_ with 150 nm thickness using chemical vapor deposition (CVD) and then coating a negative photoresist (PR) thin film. After that, the TE channel is patterned by using high-resolution electron beam lithography (EBL) due to the requirement of the dimension smaller than 200 nm. Through a development and an etching, the lower SiO_2_ layer of the TE channel is formed. (2) The same processes as the step (1) except depositing Si with 175 nm form the upper Si layer of the TE channel. (3) Finally, we fabricate the TM channel by depositing a Si layer with 625 nm and a PR thin film. After patterning the TM channel by using EBL, we proceed with a development and an etching. Note that the resolution of EBL achieves better than 5 nm in the nowadays commercial facilities. As a result, the overlay control of the TE channel is not an issue while using EBL technology. Compared with the device structures reported by others^23, 24, 26^, these PBSs also have the overlay structures. Nevertheless, the minimum dimensions of the devices^23, 24, 26^ are larger than 300 nm, thus the devices can be fabricated by a more efficient photolithography technology but the resolution of using photolithography is limited by the used wavelength (157 nm for deep UV light). Certainly, for some DC-based devices^20, 22, 25, 27–30^, they used single Si waveguide structures evading the overlay control problem, simplifying the fabrication processes and reducing the overlay deviation. In order to analyze the performance of our PBS device, we first need to determine the mode properties at the input port. The relative permittivities of Si and SiO_2_ are *ε*_*Si*_ = 12.25 and *ε*_*SiO2*_ = 2.07, respectively, at a telecommunication wavelength of *λ* = 1550 nm^36^. The mode properties can be obtained by using the boundary mode analysis of COMSOL Multiphysics, which is based on the finite element scheme. The calculated effective indices (*n*_*eff*_) of the TE and TM modes are 2.7134 and 2.9439, respectively, and the corresponding mode profiles (the *x*- and *y*- components of the electric fields) are shown in Fig. 2(a,b), respectively.

**Figure 3:**
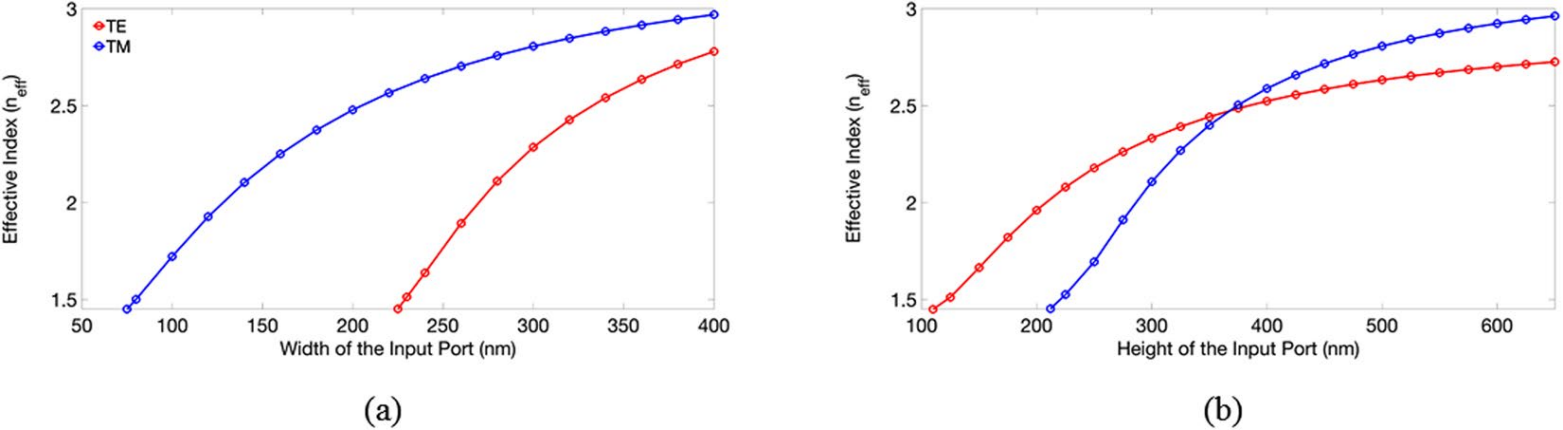
The calculated effective indices of TE and TM modes versus (**a**) the width at the height of 625 nm and (**b**) the height at the width of 380 nm of the input port.

For analyzing the mode properties, the variations of effective indices of the TE and TM modes versus the width and height of the input port are shown in Fig. 3(a,b), respectively. The cutoff widths of the TE and TM modes are 225 nm and 75 nm, respectively; and the cutoff heights of the TE and TM modes are 110 nm and 210 nm, respectively.

**Figure 4:**
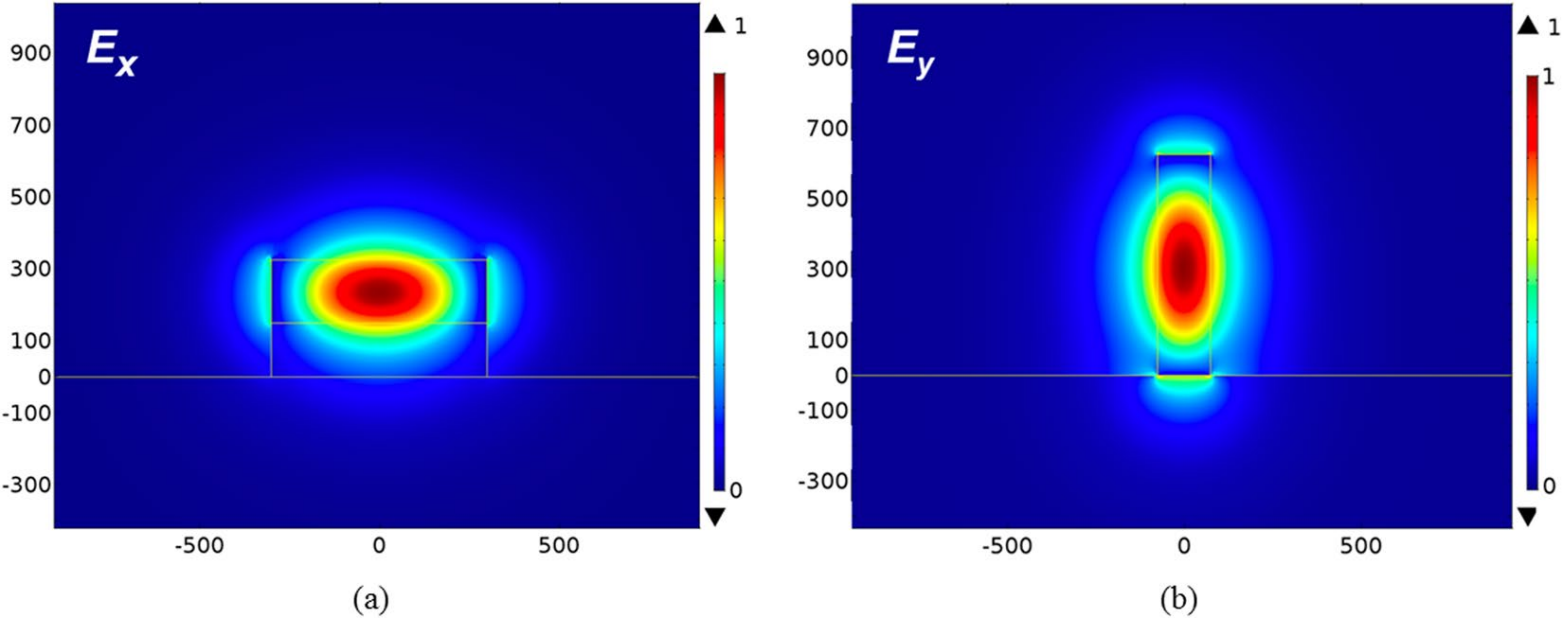
The guided mode profiles of the (**a**) TE mode (electric field component, *E*_*x*_) at the TE outport and the (**b**) TM mode (electric field component, *E*_*y*_) at the TM outport.

Note that the increasing rate of effective index versus the width of the TE mode is higher than that of the TM mode as shown in Fig. 3(a) due to the *x*-direction polarization of the TE mode. Similarly, the TM mode has higher increasing rate of effective index versus the height due to the *y*-direction polarization as shown in Fig. 3(b). In addition, to propagate the TM power but cut off the TE one along the TM channel, the width is between 75 nm and 225 nm. In contrast, the height of the TE channel is between 110 nm and 210 nm to propagate the TE power but cut off the TM one. We found that the input port supports the major electric field profiles, *E*_*x*_, of the TE mode and, *E*_*y*_, of the TM modes concentrated in the Si region. From the mode profiles, we know that a narrowing and lowering of the width and height, respectively, of the input port can enable the cut-off condition for the TE and TM modes, respectively. In order to effectively separate the two modes, the output ports of the TE and TM modes are designed to be 600 and 150 nm wide, respectively, and 175 and 625 nm tall, respectively. The designs of choosing the geometries of the TE and TM output ports are explained as follows. In Fig. 2, we observe that the TE mode profile extends moderately out of the input port along the *x*-direction, but most of the TM one is concentrated in the input port along the *y*-direction. As a result, we keep the height of the output TM port the same as the input port but increase the width of the output TE port to improve the power fraction in the TE channel. On the other hand, shrinking the width of the TM mode can effectively leak out the TE power. For trading off the optimal performances, we choose the average value ((75 + 225)/2) of the cutoff widths of the TE and TM modes as shown in Fig. 3(a). The same reason for designing the height of the TE channel, we choose the width of 175 nm for the output TE port. In particular, we add a SiO_2_ layer with 150 nm beneath the Si layer to improve the coupling efficiency of the TE mode between the input port and the TE channel. In addition, we add a SiO_2_ layer with a height of 150 nm below the TE channel in order to improve the ILs (thereby increasing the coupling efficiency between the input TE mode and the TE channel) of the TE channel. The calculated effective indices of the output TE and TM modes are 2.338 and 2.181, respectively. The difference of the effective index of the TE mode between the input port (2.7134) and the output TE port (2.338) is smaller than of the TM mode between the input port (2.9439) and the output TM port (2.1804), thus the ILs of the TE mode are expected to be lower than that of the TM mode (will be validated below) due to the better mode coupling efficiency of the TE modes. The mode profiles of the TE and TM ports are also shown in Fig. 4(a,b), respectively, for the purpose of comparison.

### Propagating performance of the designed PBS

**Figure 5:**
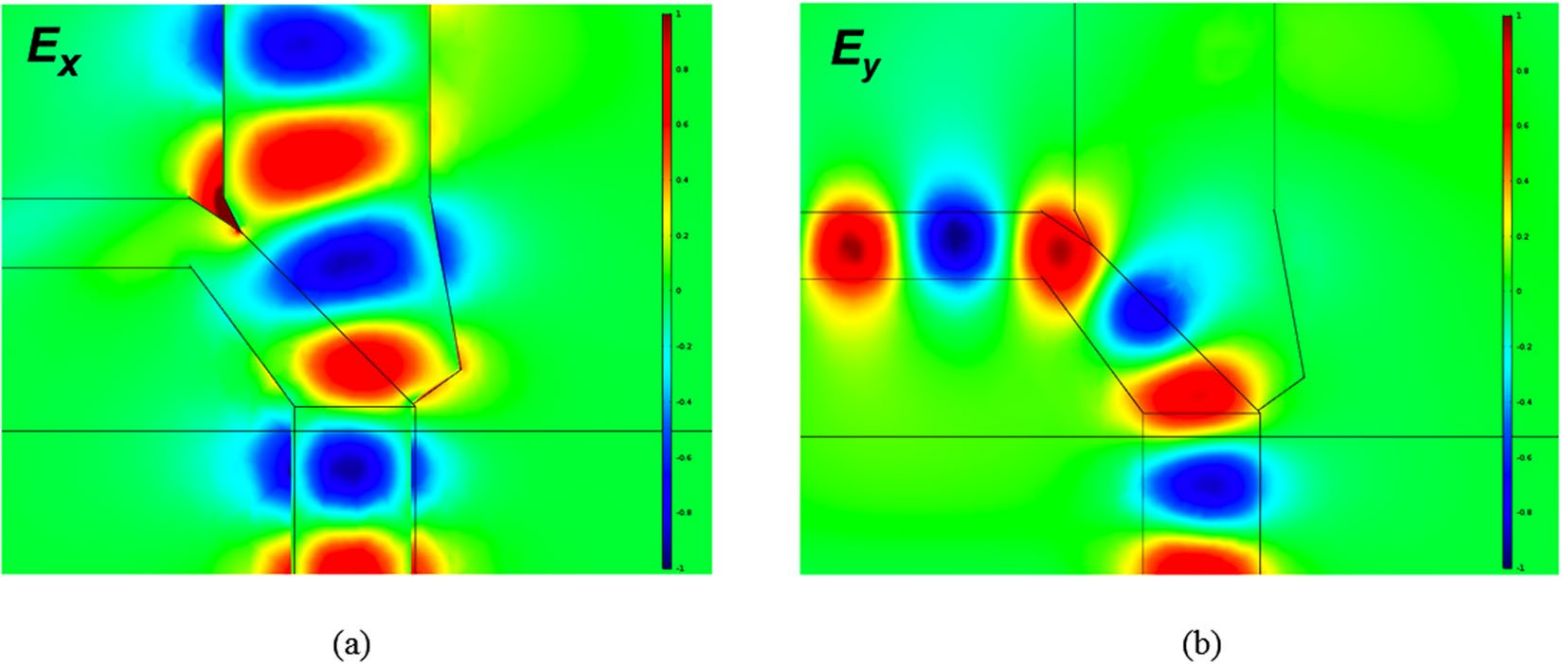
The propagating electric field distributions of the (**a**) TE (electric field component, *E*_*x*_) and (**b**) TM (electric field component, *E*_*y*_) modes.

In order to evaluate a PBS, some factors have to be considered, such as its PER, IL, size, operating bandwidth, fabrication tolerance, and geometric complexity. By launching the TE and TM modes into the input port of 625 nm × 380 nm, their propagating electric field distributions are shown in Fig. 5(a,b), respectively. We found that the input TE mode was directly coupled to the lower but wider TE channel with output port dimension of 175 nm × 600 nm. In contract, the input TM mode was guided along the tilted tapered Si waveguide to the TM channel with output port dimension of 625 nm × 150 nm. The different geometrical aspect ratios of the Si waveguides effectively separated the two polarizations depending on the supportable geometries.

**Figure 6:**
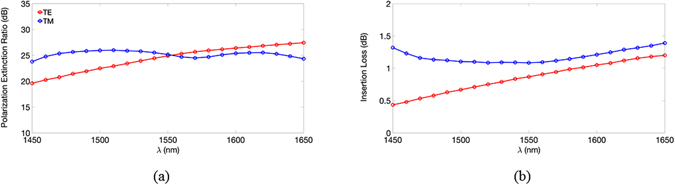
The calculated (**a**) PERs and (**b**) ILs of the TE and TM modes versus the operating wavelength, *λ*.

To precisely analyze the performance of the device, we needed to consider its PER and IL (see methods). At the telecommunication wavelength of *λ* = 1550 nm, the calculated PERs and ILs of the TE (TM) mode are 24.89 (25.16) dB and 0.87 (1.09) dB, under a volume of 790 nm (*x*) × 625 nm (*y*) × 600 nm (*z*), which is the smallest PBS with satisfactory performance designed to date. Moreover, by using a simple fabrication process and being CMOS-compatible, the designed PBS has great potential for realizing high-density PICs. In the present design, the tapered waveguide structure of the TM channel can reduce the propagating loss of the TM power and block the TE power. Likewise, the lower waveguide geometry of the TE channel avoids the TM mode from supporting within, and the wider geometry and the SiO_2_ layer below increase the coupling efficiency (thereby reducing the ILs) from the input to the TE ports, which compensates for the scattering loss due to the variation of the waveguide height. In addition to considering the PERs and ILs, we analyze the operating bandwidth of our PBS. The calculated PERs and ILs of the two polarizations versus the operating wavelength *λ* between 1450–1650 nm, considering the material dispersions of Si and SiO_2_^36^, are shown in Fig. 6(a,b), respectively. The PERs of the TE and TM modes exhibited satisfactory performance levels of >21 and 24 dB, respectively, and their ILs were <1.1 and 1.3 dB, respectively (with >77 and 75% transmittance, respectively), over a broad bandwidth of 150 nm (between 1475–1625nm).

From Fig. 6(a,b), it can be seen that the proposed PBS shows moderate wavelength-insensitivity, because of the avoidance of the phase-matched conditions that are required in conventional DC- and MMI-based PBSs; this enables the proposed PBS to be extremely short and broadband. In summary, the working principles for achieving the proposed PBS include two key ideas. First, the proposed PBS is consisted of two slanted Si waveguides with different geometrical aspect ratios adjoined side-by-side, therefore the TE and TM polarizations could be separated according to their polarization properties directly. This design avoids an additional coupling region that is often required in conventional DC-based PBSs. As a result, we can significantly reduce our PBS to the submicron scale. Second, through optimal geometry designs for the input and two output ports, the proposed PBS achieves high performances over a broad bandwidth of 150 nm. If the proposed device dimension is reduced further, we have to increase the slanted angle of the TM channel and narrow down the TE channel. Those modifications will lead to higher ILs and the lower PERs to the operating wavelength.

**Figure 7:**
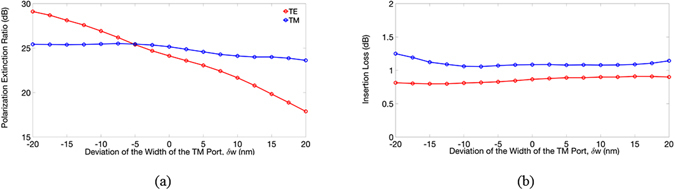
The calculated (**a**) PERs and (**b**) ILs of the TE and TM modes versus the deviation of the width of TM port, *δw*.

Following the analysis of the operating bandwidth, we then investigated our device’s fabrication tolerances. In the proposed PBS, the relatively smaller geometries were the height (175 nm) of the TE output port and the width (150 nm) of the TM output port. For the TM port, the calculated PERs and ILs of both modes versus the deviation (*δw*) of the width, *w* (*i.e*., $\overline{{GH}}$, where the point *I* is shifted up or down similarly to point *H*) are shown in Fig. 7(a,b), respectively. Within the range of *δw* = ±15 nm, the PERs and ILs of the TE (TM) mode were greater 20 (23) dB and smaller 1.0 (1.2) dB, respectively.

**Figure 8:**
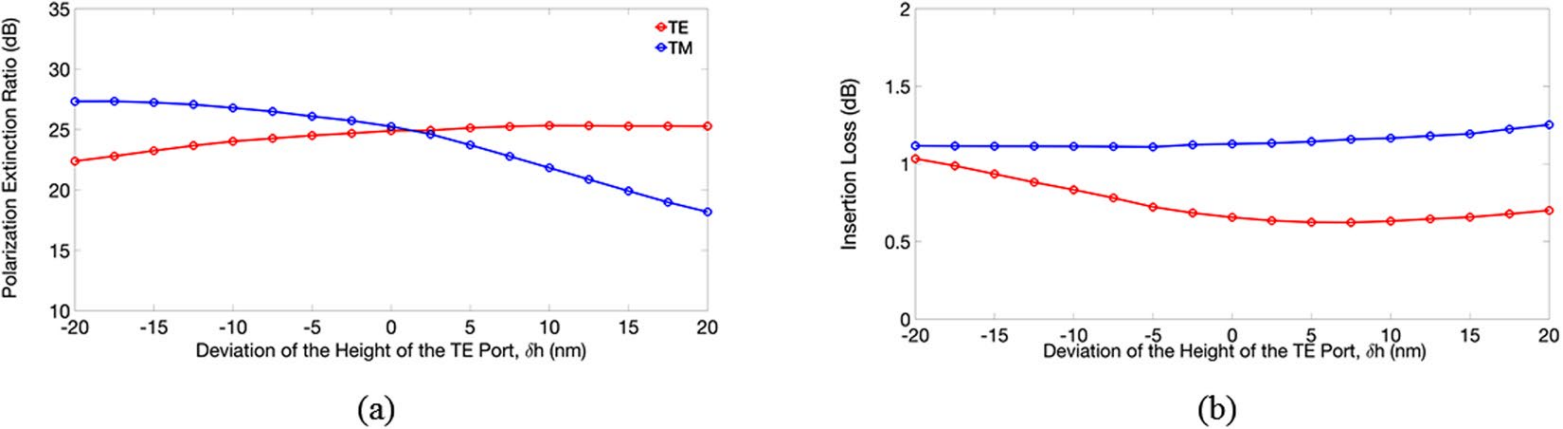
The calculated (**a**) PERs and (**b**) ILs of the TE and TM modes versus the deviation of the height of TE port, *δh*.

We observed that decreasing the width of the TM port significantly increased the PER of the TE mode, because the narrower TM port, the earlier the cut-off condition of the TE power is achieved, resulting in more TE power radiating out of the TM channel. In contrast, the ILs of the TE mode showed no significant variation, because the input TE power coupling to the TE channel was unchanged when the width of the TM port decreased. As expected, the PER and IL of the TM mode were slightly influenced by the width of the TM port. For the TE output port, the calculated PERs and ILs of both modes versus the deviation (*δh*) of the height *h* are shown in Fig. 8(a,b), respectively.

**Figure 9:**
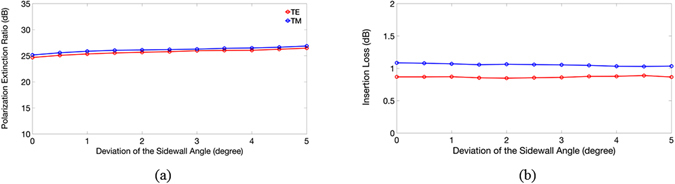
The calculated (**a**) PERs and (**b**) ILs of the TE and TM modes versus the deviation of the sidewall angles.

Within a range of *δh* = ±15 nm, the PERs and ILs of the TE (TM) mode were greater 23 (20) dB and smaller 1.0 (1.3) dB, respectively. We found that decreasing the height of the TE port significantly increased the PER of the TM mode, because lowering the height of the TE channel reduced the TM power coupling more. Similarly, the variation in the IL of the TM mode was small, because the input TM mode flowing into the TM channel did not really change as the height of the TE channel decreased. Finally, we considered the fabrication error of the etching processes. Namely, the sidewalls of the TM ($\overline{{AIH}}$) and TE ($\overline{{BCD}}$) channels were not perfectly perpendicular to the SiO_2_ surface, and so the two ports formed trapezoidal cross-sections. The calculated PERs and ILs versus the deviation angle of the sidewalls are shown in Fig. 9(a,b), respectively.

Within a 5° deviation angle, the PERs and ILs of both modes did not vary, and when the angle reached 10°, the PER and IL of the TM mode remained at around 24 and 1.3 dB (not shown), respectively. The numerical results demonstrate that the present PBS has a high fabrication tolerance.

We report on a PBS with a footprint of 600 × 790 nm^2^ using all-dielectrics of Si and SO_2_ only. The proposed PBS consisted of two slanted Si waveguides with different geometrical aspect ratios, so that the TE and TM polarizations could be separated according to their polarization properties. By avoiding the additional coupling region that is often required in conventional PBSs, we were able to reduce our PBS so that it was at the submicron scale. The device had a PER and IL of roughly 25 dB and less than 1.1 dB (which was roughly 77.7% of transmittance), respectively, while operating at a telecommunication wavelength of *λ* = 1550 nm. Over a bandwidth of 150 nm (from *λ* = 1475–1625 nm), the proposed PBS was still able to attain PERs (ILs) of greater (smaller) than 20 (1.3) dB for both TM and TE modes. Additionally, the designed PBS allowed for high fabrication tolerances of ±15 nm of the waveguide geometries and of 10° of the waveguide sidewalls while retaining a PER of around 20 dB. Our design will allow for highly dense PICs to be developed that have satisfactory levels of performance and can be fabricated using the CMOS manufacturing processes.

## Methods

In this study, for evaluating the transmission characteristics of a PBS, we need to investigate the PERs and ILs of a specific mode. Here, the TE and TM modes are defined in Eqs (1) and (2), respectively^24, 27, 29^:

1 $$PE{R}_{TE,TM}=10\,{\mathrm{log}}_{10}({P}_{TE,TM}/{P}_{TM,TE}),$$

and

2 $$I{L}_{TE,TM}=-10\,{\mathrm{log}}_{10}({P}_{TE,TM}/{P}_{input}),$$

where *P*_*i*_ is the mode power at port *i* (*i* = *TE*, *TM*, or *input*).
